# Byways of Medical Relief
*Previous articles in this series appeared in The Hospital of October 7, 21, November 18.


**Published:** 1911-12-02

**Authors:** 


### BYWAYS OF MEDICAL RELIEF.*

(Contributions are invited to this column.)

### IV.—Little Homes as a Hobby.

The little homes which receive from three to six patients are almost innumerable, and in the aggregate do far more than any one dreams to alleviate suffering. They help the hospitals most effectually by warding off approaching illness no less than by receiving persons enfeebled after illness and severe operations. As examples of private undertakings of this character may be mentioned the Convalescent Home for Girls near Compton, with four beds; and Miss Aitchison's Convalescent Cottage near Lyndhurst, with four beds. A glance through the columns of "Burdett's Annual" will reveal that these little homes under private management are in constant process of formation and disintegration. This is entirely natural and not to be regretted. They are formed and dissolved without fuss or publicity and accomplish good work during the lifetime of their founders, or for so long as opportunity offers. Even with only four beds available, as many as fifty or sixty persons may be benefited in the course of a year. The influence exercised in homes of this description, managed on common-sense lines, is very valuable in bringing classes together. Those who benefit and those who are benefited mutually learn a great deal, and these delicate strands of sympathy serve to bridge a gulf which in some countries has proved almost insurmountable. Among the many rich, generous and lonely people in our midst there are many who would find a constant source of interest in such undertakings if they could be brought to understand how easily a few convalescents can be received and made happy. Those who are drawn to this most necessary work are recommended to put themselves in communication with their nearest hospital and state what class of patients they are willing to take: men, women or children, surgical or medical. It is only where open wounds are received that a nurse need be placed in charge. Otherwise a thoroughly trustworthy cook-housekeeper, supervised by someone personally concerned in the enterprise and living close by, suffices. A six-roomed cottage, with two airy bedrooms for the patients, a living-room, kitchen, and accommodation for the housekeeper, is all that is needed for three or four cases. The matron of the hospital can be relied on to select suitable patients, and an immense support is afforded by an understanding that in the event of any being sent who from the nature of their complaints or their unsatisfactory character, prove undesirable inmates, they can be sent back to the hospital. The local medical practitioner should be invited to visit the patients on arrival and prescribe, if necessary, and should receive a fixed yearly sum for his services. It is advisable in these and similar cases to avoid the smallest shadow of doing good at the expense of other people.

The scope of the little home of this description might easily be extended to embrace chronic sufferers such as often in great towns linger out their days in a kind of living death in some stuffy room where no ray of hope or joy ever penetrates to them. Often these sufferers need merely a happier environment to bring them new vigour. But whether they eventually prove curable or no, it is certain that it is very Christ-like work to bring them hope in the midst of despair and minister to minds and bodies wasted from neglect.

From the outset the advantages of an open-air life should be kept firmly in view. Many chronic sufferers have an unreasonable dread of the merest breath of air. They must not be made miserable by exposure to draughts before they have learned to appreciate their life-giving qualities. However small the garden space available it will be found that screens securely fixed at right angles to each other in the form of a cross in the centre of the recreation ground will afford two sheltered spaces in whatever direction the wind may be blowing. These screens or fences may be gradually planted up with roses and creepers and turned into an agreeable feature of the home.

The mental condition of chronic patients is of the first importance. However suffering the patient may be he should be induced to take an interest in some easy occupation, preferably for the benefit of another. Training the fingers to undertake some interesting craft, assisting patients to make something and thus taste the pleasures of achievement: these should be considered essential parts of the treatment. Money spent on securing a few lessons for the patient in arts and crafts suited to their infirmities brings in a richer reward in the wave of increased vitality it sets stirring than costly luxuries soon partaken of and forgotten. The managers of these little homes may always count upon generous assistance in the task of making life bearable to those under their care, and lessons would be as readily contributed as grapes were the need realised.

But when good accommodation, good nursing, fresh air, and interesting occupations have been secured, the chronic patients will still imperatively need a rich blend of faith, hope and charity in those who are responsible for their welfare.

Let there be no mistake about it. They will need all the patience, all the faith in human nature, all the treasures of hope and vitality which can be poured out at their feet. For many weeks, it may be, their benefactor will need to give pressed down and running over, expecting nothing in return, before the crust of complaining and dissatisfaction with life begins to melt. But the harvest is sure. If the little children yield the quickest, sweetest fruits to those who tend them, the slowly ripening gratitude of those who in later life feel the gladdening effects of true service holds more lasting joy.

* Previous articles in this series appeared in The Hospital of October 7, 21, November 18.

